# Supplementary material for: Molecular genetic diversity of seaweeds morphologically related to Ulva rigida at three sites along the French Atlantic coast
Source: PeerJ. 2021 Dec 24;9:e11966. doi: 10.7717/peerj.11966 (PMC8711279; doi:10.7717/peerj.11966)
Supplement: Supplemental Information 1 — *: Additional rbcL sequence. [file peerj-09-11966-s001.docx]

Supplementary S1: Accession numbers and description of *tufA* sequences deposited in Genbank. *: Additional *rbcL* sequence.

| Accession number | Species | Haplotype | Site | Collectors | Date of collection | Initial length |
| --- | --- | --- | --- | --- | --- | --- |
| MT078946 | *U. rigida / U. laetevirens* | H1 | Roscoff (France) | Dartois M., Aubert F., Huet V., Prineau M., Sauriau P.-G. | Feb-2019 | 850 bp |
| MT078947 | *U. rigida / U. laetevirens* | H1 | Concarneau (France) | Dartois M., Aubert F., Huet V., Prineau M., Sauriau P.-G. | Feb-2019 | 858 bp |
| MT078948 | *U. rigida / U. laetevirens* | H1 | La Tranche sur Mer (France) | Dartois M., Aubert F., Huet V., Prineau M., Sauriau P.-G. | Feb-2019 | 877 bp |
| MT078949 | *U. rigida / U. laetevirens* | H2 | Roscoff (France) | Dartois M., Aubert F., Huet V., Prineau M., Sauriau P.-G. | Feb-2019 | 864 bp |
| MT078950 | *U. rigida / U. laetevirens* | H2 | Concarneau (France) | Dartois M., Aubert F., Huet V., Prineau M., Sauriau P.-G. | Feb-2019 | 876 bp |
| MT078951 | Undetermined species | H3 | Concarneau (France) | Dartois M., Aubert F., Huet V., Prineau M., Sauriau P.-G. | Feb-2019 | 858 bp |
| MT078952 | *U. australis* | H4 | Roscoff (France) | Dartois M., Aubert F., Huet V., Prineau M., Sauriau P.-G. | Feb-2019 | 807 bp |
| MT078953 | *U. australis* | H4 | Concarneau (France) | Dartois M., Aubert F., Huet V., Prineau M., Sauriau P.-G. | Feb-2019 | 815 bp |
| MT078954 | U. fenestrata | H5 | Concarneau (France) | Dartois M., Aubert F., Huet V., Prineau M., Sauriau P.-G. | Feb-2019 | 858 bp |
| MT078955 | *Ulva armoricana* Dion, de Reviers & Coat (MNHN-PC0115137) |  | Roscoff (France) | Coat C., Dion C., Noailles C., Berger Y. | 1996 | 864 bp |
| MT078956 | *Ulva scandinavica* Bliding (MNHN-PC0547277) |  | Saint-Brieuc (France | Kuhlenkamp R. | 1996 | 872 bp |
| MT078957 | *Ulva rigida* C. Agardh var. *fimbriata* J. Agardh (MNHN-PC0531492) |  | La Coruna (Spain) | Pérez-Cinerea J.L., Cremades J., Barbara I. | 1989 | 862 bp |
| MW013545* | *Ulva pseudorotundata* | H3 | Concarneau (France) | Dartois M., Aubert F., Huet V., Prineau M., Sauriau P.-G. | Feb 2019 | 238 bp |
