# Supplementary figures and images for: Molecular genetic diversity of seaweeds morphologically related to Ulva rigida at three sites along the French Atlantic coast
Source: PeerJ. 2021 Dec 24;9:e11966. doi: 10.7717/peerj.11966 (PMC8711279; doi:10.7717/peerj.11966)

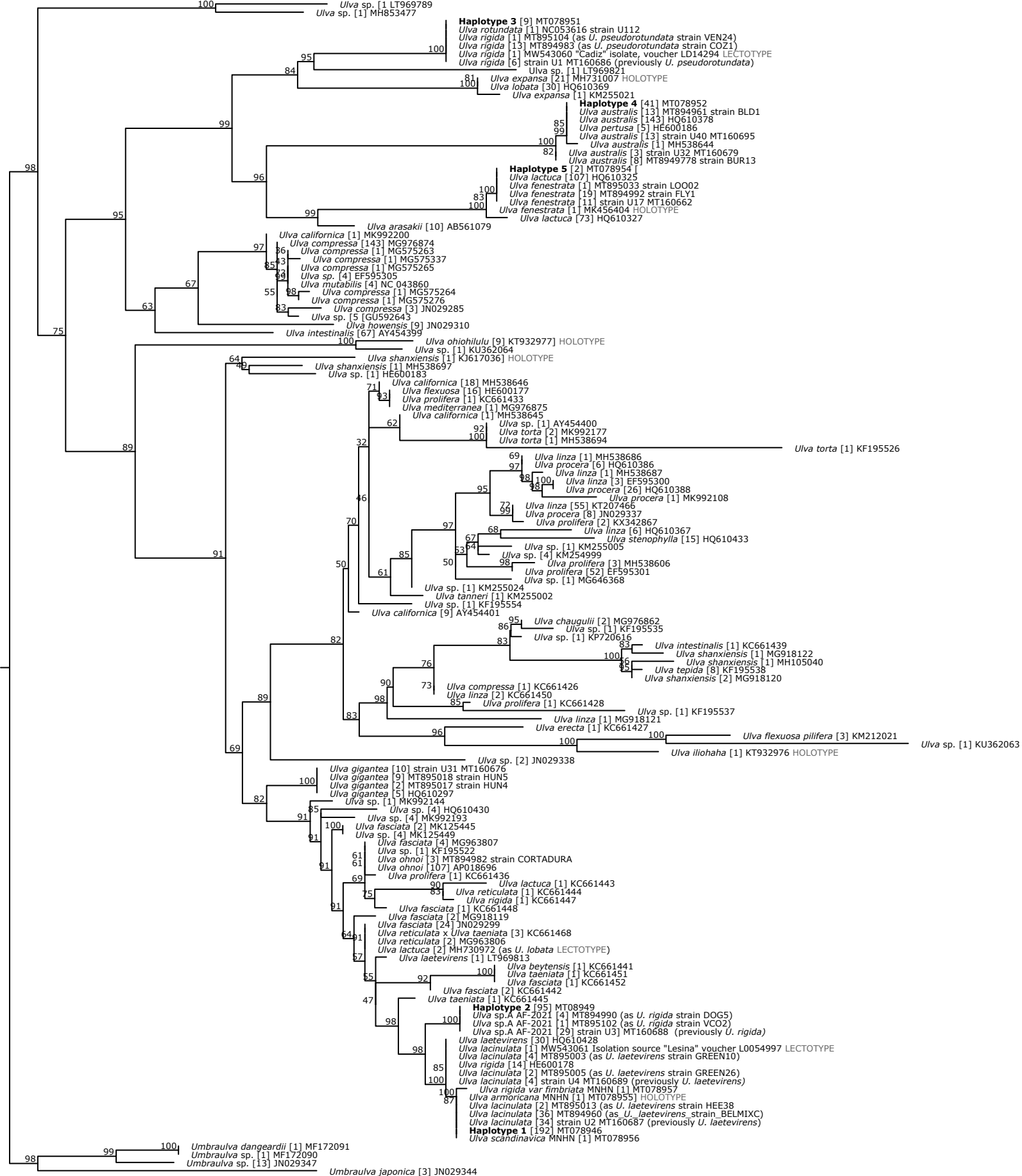

0.02

Supplement: Supplemental Information 2 — Haplotypes detected in this study are in bold. Bootstrap support values from the ML analysis are indicated on each internal branch. Sample size is presented after the haplotype name. Unit of scale bar: substitution/site. MNHN: Muséum National d’Histoire Naturelle, Paris. [file peerj-09-11966-s002.pdf]

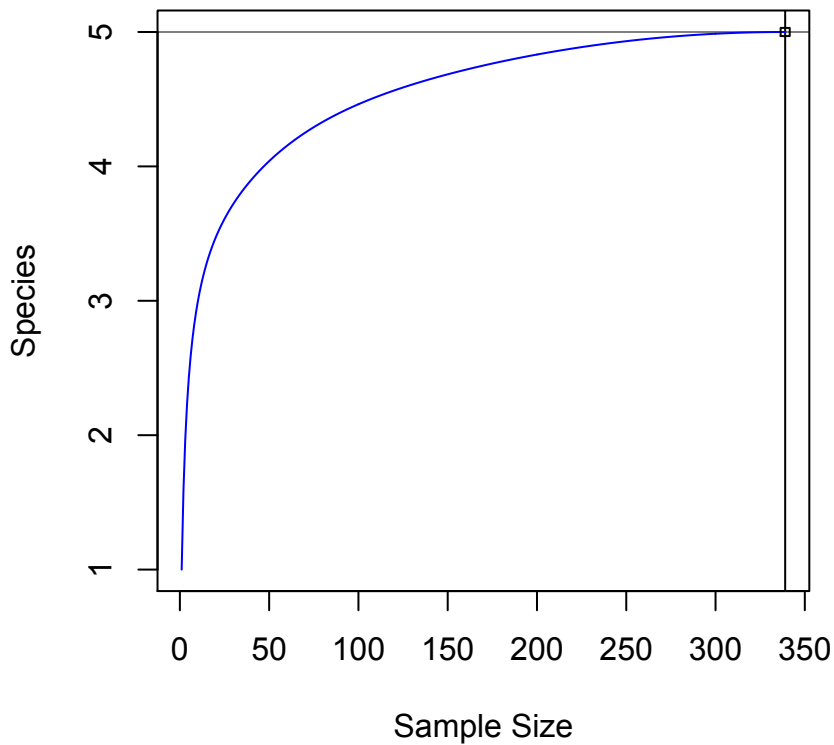

Supplement: Supplemental Information 4 [file peerj-09-11966-s004.pdf]

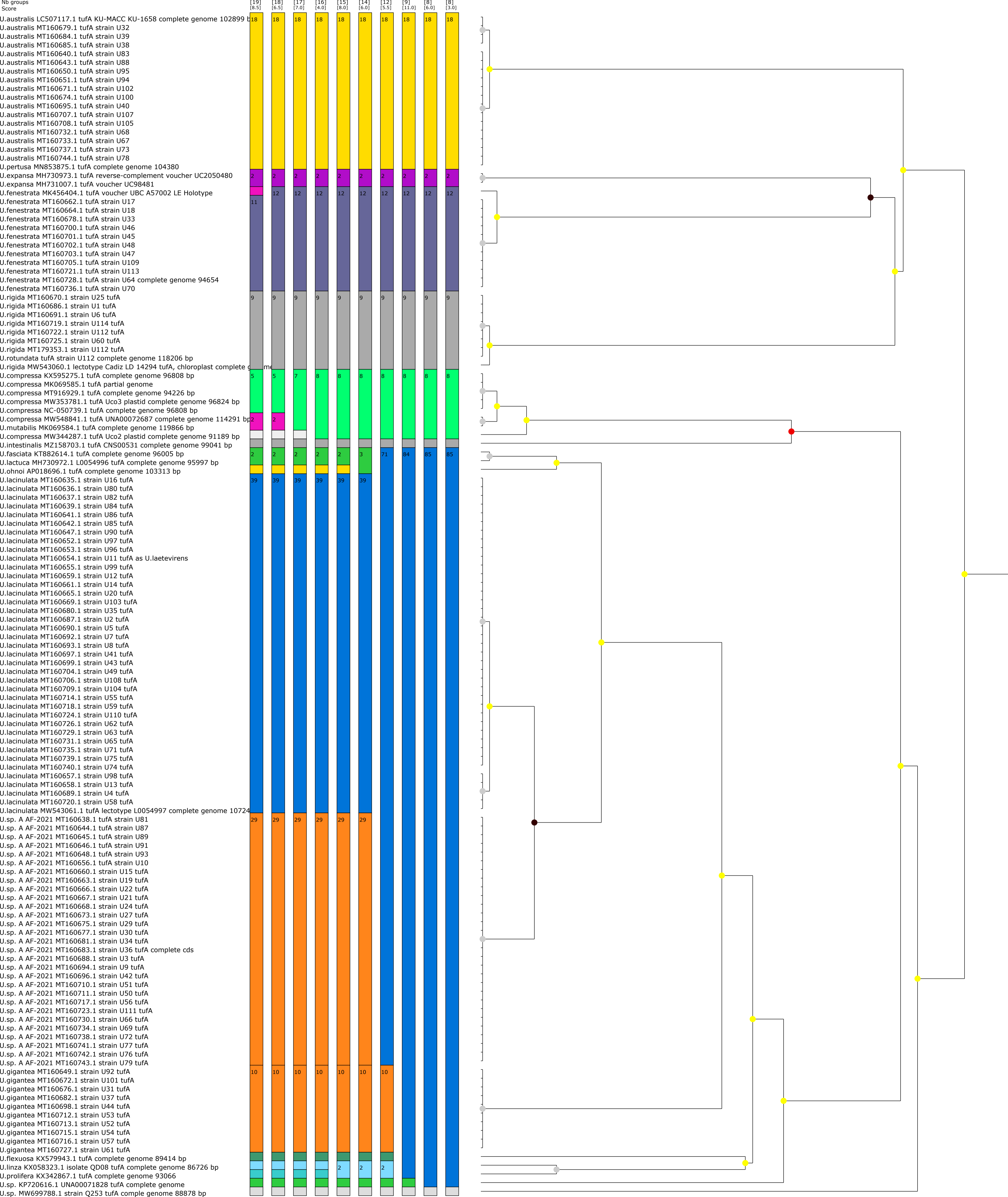

Supplement: Supplemental Information 9 — Based on 136 sequences of Ulva for the chloroplastic gene tufA (1224 bp). [file peerj-09-11966-s009.pdf]
